# Miniaturized GPS Tags Identify Non-breeding Territories of a Small Breeding Migratory Songbird

**DOI:** 10.1038/srep11069

**Published:** 2015-06-09

**Authors:** Michael T. Hallworth, Peter P. Marra

**Affiliations:** 1 Migratory Bird Center, Smithsonian Conservation Biology Institute, National Zoological Park, MRC 5503, Washington DC 20013.

## Abstract

For the first time, we use a small archival global positioning system (GPS) tag to identify and characterize non-breeding territories, quantify migratory connectivity, and identify population boundaries of Ovenbirds (*Seiurus aurocapilla*), a small migratory songbird, captured at two widely separated breeding locations. We recovered 15 (31%) GPS tags with data and located the non-breeding territories of breeding Ovenbirds from Maryland and New Hampshire, USA (0.50 ± 0.15 ha, mean ± SE). All non-breeding territories had similar environmental attributes despite being distributed across parts of Florida, Cuba and Hispaniola. New Hampshire and Maryland breeding populations had non-overlapping non-breeding population boundaries that encompassed 114,803 and 169,233 km^2^, respectively. Archival GPS tags provided unprecedented pinpoint locations and associated environmental information of tropical non-breeding territories. This technology is an important step forward in understanding seasonal interactions and ultimately population dynamics of populations throughout the annual cycle.

Current knowledge of when and where migratory animals go throughout their annual cycle, i.e. migratory connectivity, is highly limited^1^. Yet this information is urgently needed given that many songbird species are exhibiting significant and enigmatic population declines^2^. Understanding how animals are distributed through space and time improves our ability to quantify the processes governing the dynamics of migratory populations. Unfortunately, identifying locations of individuals within a population during subsequent periods of the annual cycle, such as on the non-breeding grounds with high spatial resolution, has been only possible with large-bodied species (e.g., raptors, seabirds).

Until recently, our ability to track animals across space and time was limited to platform transmitting terminals (PTT; satellite transmitters) or similar devices like GSM (Groupe Spécial Mobile) tags. PTT tags use Argos satellites to determine geolocations at 1 km resolution and can provide information into broad scale habitat use^3^, migration timing and strategies^4^ as well as local movements during stationary periods^5^. However, their size (>5 g) restricts their use to large-bodied reptiles^6^, birds^3^ and mammals^7^ and precludes their use on small organisms. GSM tags use Global Positioning Systems (GPS) technology and can provide location data with fine scale resolution (~meters) by simultaneously calculating the distance from multiple NAVSTAR GPS satellites for spatial location fixes. Data are then transmitted to users via cellular networks^8^. Unfortunately, GSM devices are heavy (18-70 g) and thus are restricted to larger animals. Both PTT and GSM tags are also expensive (>$3000 each) which may prohibit large-scale projects.

Tracking small migratory organisms (<20 g) is possible with the miniaturization of archival light-level geolocators^9^ (hereafter geolocators), however limitations exist, including the need to recover the devices to obtain the data. Spatial resolution in location estimates is large (latitude: ~200 km, longitude: ~150 km), especially for forest dwelling species^10^, and latitudinal estimates are unreliable either side of vernal and autumnal equinox, which coincides with spring, fall or both migratory movements^11^,^12^. Therefore, light-level geolocators are limited by their inability to provide high resolution and precise location data. For high-resolution spatial tracking, GPS technology is required.

Here we tested and deployed a newly developed miniaturized tracking device that weighs ~1 g, combines archival technology with GPS accuracy (~10 m) and can be placed on small songbirds weighing <20 g. Until now, the smallest comparable device available was ~12 g and could only be placed on animals weighing at least 250 g^13^. Given the accuracy of the location data, GPS tags placed on breeding birds can provide high-resolution locality information from non-breeding territories, migratory routes and migration timing depending on flexible programming. In this paper, we provide the first data from miniaturized archival GPS tags deployed on a small (<20 g) migratory songbird. First, we identify individual non-breeding territories and then characterize and compare their environmental attributes using remote sensing. Second, we define non-breeding season population boundaries for our two distinct breeding populations and quantify the strength of migratory connectivity.

## Results

Twenty-four tagged Ovenbirds (*Seiurus aurocapilla*) returned to breed in 2014 (Hubbard Brook Experimental Forest, NH (HBEF): n = 10, 42%; Jug Bay Wetland Sanctuary, MD USA (JBWS): n = 14, 58%). Six individuals (HBEF: n = 4, JBWS: n = 2) returned without the GPS tag, the antenna fell off two tags resulting in failure to acquire data (JBWS: n = 2) and one tag did not function properly (HBEF: n = 1), resulting in location data from 15 individuals (HBEF: n = 5, JBWS: n = 10; Table S2).

**Figure 1:**
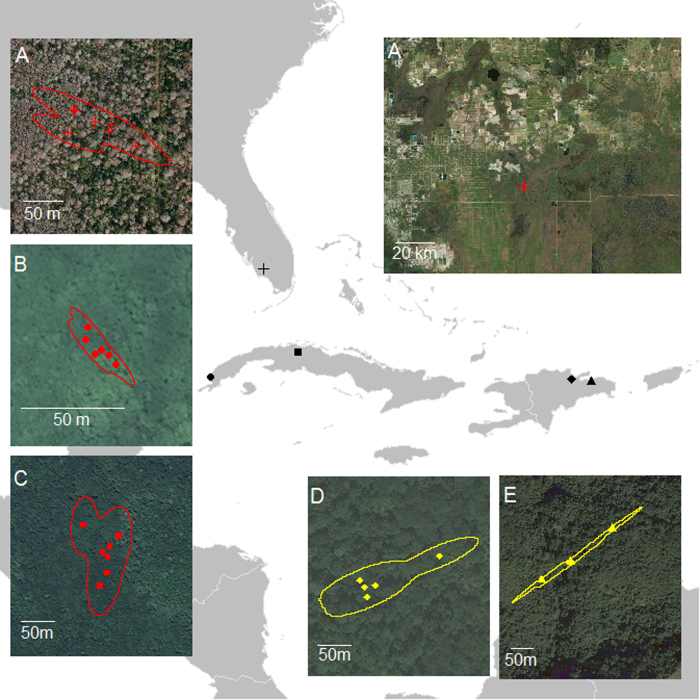
Example of five Ovenbird non-breeding territories captured in Maryland (JBWS: red **A**-**C**) and New Hampshire (HBEF: yellow **D** & **E**), United States determined using archival GPS tags. The non-breeding locations of individuals and their corresponding territories (**A**-**E**) are shown using different symbols. The 95% kernel density estimate around the location data is also shown. Maps depicted in Fig. 1 were created in program R. Inset maps are OpenStreetMap© images (www.openstreetmap.org/copyright; https://creativecommons.org/licenses/by-sa/2.0/) accessed through program R^25^. No changes were made to the original images.

Ovenbirds occupied small non-breeding territories (range 0.03 – 1.86 ha) throughout the non-breeding season (Fig. 1; Table S2). The non-breeding territory size for individuals breeding at HBEF and JBWS were 0.40 ± 0.14 (mean ± SE) and 0.52 ± 0.19 ha, respectively. Ovenbirds breeding at HBEF migrated further (2767.88 km, CI 2697.02–2838.95) than individuals breeding at JBWS (1588.32 km CI 1527.53–1648.98) with a mean difference of 1179.55 km (CI 1086.61–1271.91) between the populations. Of the environmental attributes remotely-sensed, only elevation differed between the populations (Table 1). The land cover classifications of non-breeding territories^14^ for HBEF individuals were composed of evergreen broadleaf forest (n = 2), cropland/natural vegetation mosaics (n = 2) and croplands (n = 1). Similarly, the non-breeding territories of JBWS individuals were dominated by cropland/natural vegetation mosaics (n = 6), woody savannas (n=3) and permanent wetlands (n = 1).

**Figure 2:**
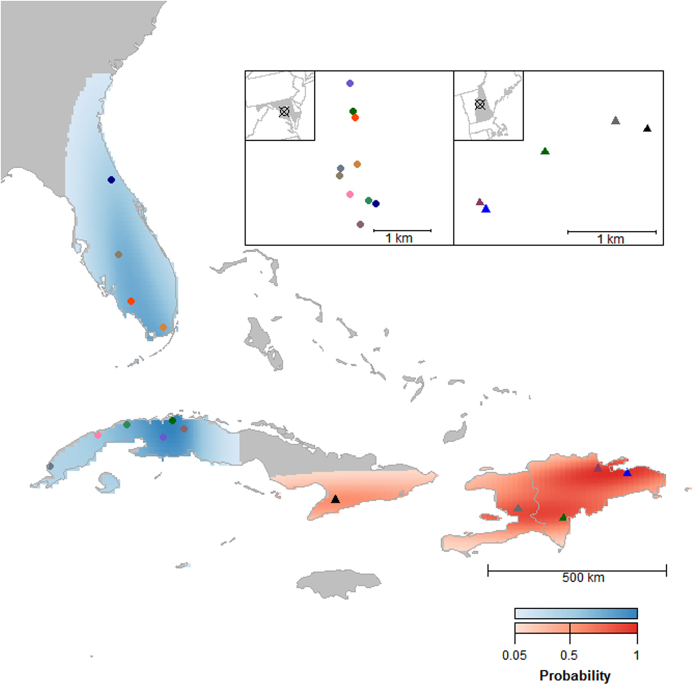
Non-breeding territories and population boundaries of Ovenbirds breeding in New Hampshire (HBEF; triangles) and Maryland (JBWS; closed circles), United States. Color ramps represent the population boundaries, the 95% kernel density estimate around non-breeding locations (HBEF = red, JBWS = blue). Individual breeding territories are shown in different colors to illustrate how individuals are spatially arranged throughout the year. The map depicted in Fig. 2 was created in program R^25^.

The non-breeding population boundary of Ovenbirds from HBEF (breeding area: 2.06 km^2^) and JBWS (15.10 km^2^) were non-overlapping and covered 114,803 and 169,233 km^2^, respectively (Fig. 2). The Mantel correlation coefficient (r_M_ = 0.839, n = 15, *P* = 0.0003) indicated that Ovenbirds exhibit strong migratory connectivity between breeding and non-breeding seasons. Individuals breeding at HBEF (r_M_ = 0.589, n = 5, *P* = 0.019) spent the non-breeding season in eastern Cuba (n = 1) Dominican Republic (n=3) and Haiti (n = 1), and had stronger connectivity than individuals breeding at JBWS (r_M_ = −0.116, n = 10, *P* = 0.592) which wintered in Florida (n = 4) and western Cuba (n = 6, Fig. 2).

## Discussion

Here, using a new miniaturized archival GPS technology we locate, with high resolution, the non-breeding territories of individual migratory songbirds captured during the breeding season. Doing so also allows us to use remotely sensed data to characterize the environmental attributes and conditions of these non-breeding territories, delineate population boundaries and quantify the strength of migratory connectivity of these two different breeding populations. Data at this resolution throughout the annual cycle were previously unattainable for species anywhere close to this size. Eighty-three percent of the recovered units (15 of 18) contained location data and obtained at least two locations from the non-breeding season. Because location data are downloaded as geographic coordinates there is also no analysis subjectivity or significant data processing time, a problem inherent within light-level geolocators^9^.

Until now it has been impossible to obtain territory-level non-breeding season locations or specific habitat information of breeding migratory songbirds remotely because the spatial resolution of light-level geolocators and stable isotopes is low (100–1000 km; but 15). Non-breeding location data for Ovenbirds analogous to those obtained with GPS tags (~10 m) would involve weeks of costly, labor intensive field work^16^ and the breeding origin of these individuals would be unknown. Territory sizes identified with GPS tags remotely are consistent with Ovenbird non-breeding territories determined in the field with radio telemetry in Belize^17^ and Jamaica^16^ confirming this as a viable option for estimating territories remotely. In addition, these new GPS tags provide location data from potentially physically and/or politically (i.e., Cuba) difficult to access locations. Therefore, the combination of remotely sensed data with simultaneous, unbiased location data provided by GPS tags signifies a major advance for the study of small migratory animals. The fact that they need to be retrieved remains a disadvantage.

The non-breeding population boundaries of our two breeding populations were non-overlapping and encompassed four different political boundaries (Cuba, Dominican Republic, Haiti and United States). Delineating population boundaries throughout the annual cycle is an important advance as it allows for the integration of demographic rates into dynamic models for linked populations^18^, necessary to identify processes that limit or regulate populations and determine when and where they operate. Interestingly, we identified the non-breeding population boundary of this same breeding population at HBEF using light-level geolocators^12^. Although the non-breeding area characterized by geolocators was large (231,509 km^2^), the population boundaries for HBEF we identify here was entirely encapsulated (100%-114,803 km^2^ of 231,509 km^2^) within estimates derived with light-level geolocators confirming that these new population boundaries are robust. Despite having small sample sizes, the non-breeding population boundary size for JBWS reached an asymptote around 10 individuals (Fig. S2). Identifying linked breeding and non-breeding populations across the range of a species will enable assessments of how past changes in land use and climate influence population trends as well as make predictions about the potential impacts of future environmental stressors.

The strength of migratory connectivity we quantify here using the Mantel approach^19^,^20^ was surprisingly strong, although our sample sizes were small and from only two locations. Additional research using tracking devices that provide high-resolution spatial data, along with large sample sizes, will likely provide novel insights into our understanding of migratory connectivity from this and other small-bodied migratory species. Such data will improve our understanding of the role that varying strengths of migratory connectivity play in driving seasonal interactions, delineating population boundaries and ultimately in population dynamics.

The high-resolution tracking of a small migratory songbird, from a breeding to a non-breeding territory at 10 m accuracy, is a major step forward for understanding the ecology of small migratory animals. Continued improvements in this technology will further increase our ability to answer complex questions with regard to seasonal interactions and population dynamics and hopefully help in our ability to conserve migratory species.

## Methods

We deployed archival GPS tags on Ovenbirds, a small (~20 g) long-distance migratory songbird, breeding within Hubbard Brook Experimental Forest, NH (HBEF; 43.94° N, −71.73° W) and Jug Bay Wetland Sanctuary, MD USA (JBWS; 38.77° N, −76.69° W) during the 2013 breeding season (May-June). At each site, we attached a PinPoint-10 archival GPS tag (1.1 g, Lotek Wireless Inc.) on twenty-four males weighing >20.0 g. Tags were programmed to begin collecting data on 1 July 2013 with a delay of 28 days (maximum delay period possible at time of deployment) between location estimates to maximize the likelihood of obtaining non-breeding location data. All tags were programmed to collect data on the same schedule (see supplemental information). The following year, individuals that returned to breed were recaptured (April and May 2014), devices were removed and data downloaded (PinPoint software, Lotek Wireless Inc.). Location data are downloaded as geographic coordinates therefore no further processing is needed to determine locations.

### Identifying and Characterizing Attributes of Non-breeding Territories.

Individual territories were constructed using the location data during the stationary non-breeding season (see Supplemental Information). Environmental attributes of non-breeding territories were quantified using remotely sensed data (Table S1) annotated to each location using Env-DATA Track Annotation Service from movebank.org^5^.

### Delineating population boundaries and Quantifying Migratory Connectivity.

We used location data from the GPS tags to delineate relative boundaries for each breeding population. We define boundaries during the non-breeding season as the 95% kernel density estimate around the non-breeding locations for each breeding population^7^. We quantified the strength of migratory connectivity using the Mantel correlation coefficient (*r*_M_)^19^,^20^. Values close to 1 indicate a strong correlation between breeding and non-breeding distance matrices (i.e., individuals breeding in close proximity also winter in close proximity).

The methods were carried out in accordance with the approved federal guidelines for using animals as research subjects. The procedures used to capture and handle Ovenbirds, and attach GPS tags were approved by the National Zoological Park’s Institutional Animal Care and Use Committee (NZP-IACUC # 11–13 and NZP-IACUC # 14–21).

## Additional Information

**How to cite this article**: Hallworth, M. T. and Marra, P. P. Miniaturized GPS Tags Identify Non-breeding Territories of a Small Breeding Migratory Songbird. *Sci. Rep*. **5**, 11069; doi: 10.1038/srep11069 (2015).

## Supplementary Material

Supplementary Information

## Figures and Tables

**Table 1:** Non-breeding territory attributes of Ovenbirds breeding in New Hampshire (HBEF) and Maryland (JBWS), United States. The mean and 95% CI of each attribute are shown. Difference values indicate the difference between the sample populations (mean and 95% CI). Bold font indicates a significant difference between the two populations.

**Non-breeding territory attributes**	**Hubbard Brook Exp. Forest, NH n = 5**	**Jug Bay Wetland Sanctuary, MD n = 10**	**Difference**
*Physical*			
Elevation (m)	282.66 (195.68 : 367.88)	38.75 (16.33 : 45.47)	**243.91 (154.61 : 332.13)**
Distance to Coast (km)	17.27 (7.26 : 28.46)	24.40 (10.08 : 39.33)	7.13 (−10.94 : 24.70)
High Vegetation Cover (%)	0.296 (0.08 : 0.56)	0.270 (0.13 : 0.41)	0.026 (−0.32 : 0.22)
*Environmental*			
NDVI – March	0.64 (0.38 : 0.89)	0.63 (0.57 : 0.69)	0.007 (−0.26 : 0.26)
NDVI – Difference Oct-March	-0.10 (−0.26 : 0.06)	-0.095 (-0.18 : −0.01)	0.005 (−0.18 : 0.18)
Air Temperature (°C)	23.54 (18.16 : 28.82)	24.23 (23.35 : 25.10)	0.68 (−4.61 : 6.02)
Soil Temperature (°C)	25.19 (19.97 : 30.53)	25.72 (24.67 : 26.75)	0.53 (−4.80 : 5.82)

## References

[b1] Webster M. S., Marra P. P., Haig S. M., Bensch S. & Holmes R. T. Links between worlds: unraveling migratory connectivity. Trends in Ecology & Evolution 17, 76–83 (2002).

[b2] Faaborg J. . Long-term decline of a winter-resident bird community in Puerto Rico. Biodiversity and Conservation 22, 63–75 (2013).

[b3] Wood A. G., Naef-Daenzer B., Prince P. A. & Croxall J. P. Quantifying habitat use in satellite-tracked pelagic seabirds: application of kernel estimation to albatross locations. Journal of Avian Biology 31, 278–286 (2000).

[b4] Limiñana R., Romero M., Mellone U. & Urios V. Mapping the migratory routes and wintering areas of Lesser Kestrels *Falco naumanni*: new insights from satellite telemetry. Ibis 154, 389–399 (2012).

[b5] Dodge S. . The environmental-data automated track annotation (Env-DATA) system: linking animal tracks with environmental data. Movement Ecology 1, (2013). 10.1186/2051-3933-1-3 PMC4337772 25709817

[b6] James M. C., Andrea Ottensmeyer C. & Myers R. A. Identification of high-use habitat and threats to leatherback sea turtles in northern waters: new directions for conservation. Ecology letters 8, 195–201 (2005).

[b7] McLoughlin P. D. . Population delineation of barren-ground Grizzly bears in the central Canadian arctic. Wildlife Society Bulletin 30, 728–737 (2002).

[b8] Bridge E. S. . Technology on the move: recent and forthcoming innovations for tracking migratory birds. BioScience 61, 689–698 (2011).

[b9] Bridge E. S. . Advances in tracking small migratory birds: a technical review of light-level geolocation. Journal of Field Ornithology 84, 121–137 (2013).

[b10] Fudickar A. M., Wikelski M. & Partecke J. Tracking migratory songbirds: accuracy of light-level loggers (geolocators) in forest habitats. Methods in Ecology and Evolution 3, 47–52 (2012).

[b11] Stanley C. Q., MacPherson M., Fraser K. C., McKinnon E. A. & Stutchbury B. J. M. Repeat tracking of individual songbirds reveals consistent migration timing but flexibility in route. PLoS ONE 7, e40688 (2012). 22848395 10.1371/journal.pone.0040688 PMC3405083

[b12] Hallworth M. T., Sillett T. S., Van Wilgenburg S. L., Hobson K. A. & Marra P. P. Migratory connectivity of a neotropical migratory songbird revealed by archival light-level geolocators. Ecological Applications 25, 336–347; 10.1890/14-0195 (2015). 26263658

[b13] Bouten W., Baaij E. W., Shamoun-Baranes J. & Camphuysen K. C. J. A flexible GPS tracking system for studying bird behaviour at multiple scales. J Ornithol 154, 571–580 (2012).

[b14] Channan S. K., Collins K. & Emanuel W. R. Global mosaics of the standard MODIS land cover type data. University of Maryland and the Pacific Northwest National Laboratory, College Park, Maryland, USA. (2014).

[b15] Fraser K. C. . Continent-wide tracking to determine migratory connectivity and tropical habitat associations of a declining aerial insectivore. Proc. R. Soc. B 279, 4901–4906 (2012). 10.1098/rspb.2012.2207 PMC3497251 23097508

[b16] Brown D. R. & Sherry T. W. Solitary winter roosting of Ovenbirds in core foraging area. The Wilson Journal of Ornithology 120, 455–459 (2008).

[b17] Kresnik R. J. & Stutchbury B. J. M. Space-use strategies of wintering Ovenbirds in Belize: causes and consequences. J. Field Ornithol. 85, 274–288 (2014).

[b18] Hostetler J. A., Sillett T. S. & Marra P. P. Full annual-cycle population models for migratory birds. The Auk: Ornithological Advances. (In press).

[b19] Ambrosini R., Møller A. P. & Saino N. A quantitative measure of migratory connectivity. Journal of Theoretical Biology 257, 203–211 (2009). 19108778 10.1016/j.jtbi.2008.11.019

[b20] Stanley C. Q. . Connectivity of Wood Thrush breeding, wintering, and migration sites based on range-wide tracking. Conservation Biology 29, 164–174 (2014). 25052795 10.1111/cobi.12352

