# Supplementary material for: Miniaturized GPS Tags Identify Non-breeding Territories of a Small Breeding Migratory Songbird
Source: Sci Rep. 2015 Jun 9;5:11069. doi: 10.1038/srep11069 (PMC4460897; doi:10.1038/srep11069)
Supplement: Supplementary Information [file srep11069-s1.pdf]

Miniaturized GPS Tags Identify Non-breeding Territories of a Small Breeding Migratory Songbird

Michael T. Hallworth[1,2], and Peter P. Marra[1]

[1]*Migratory Bird Center, Smithsonian Conservation Biology Institute, National Zoological Park, MRC 5503, Washington DC 20013*

[2]corresponding author: mhallwor@masonlive.gmu.edu

SUPPLEMENTAL MATERIAL

SUPPLEMENTAL MATERIAL

METHODS

Pin-point 10 GPS tags

Pin-point 10 GPS tags were attached to individuals using a leg harness technique [21]. Archival GPS tags were deployed on males to increase the likelihood of recapture during the following breeding season as they exhibit high site-fidelity and respond strongly to playback [22]. Location estimates are taken once satellite reception is acquired. However, if the device does not acquire satellites after 70 seconds of trying the device fails to take a location and returns to an idol state until the next scheduled interval. Prior to deployment, archival GPS tags were programmed to begin collecting data on 1 July 2013 with a delay of 28 days (maximum delay period possible at time of deployment) between location estimates to maximize the likelihood of obtaining non-breeding location data. The archival GPS tags were scheduled to take location fixes in the middle of the night (range 23:27-01:45 eastern time) so individuals would be stationary (i.e. roosting) to increase the likelihood of obtaining a satellite signal.

Attributes of Non-breeding Territories

Individual territories were constructed using all location data obtained during the stationary non-breeding season. We defined individual territories as the 95% kernel density estimate (KDE) around the location data. Brown and Sherry (2008) found that Ovenbird roosting behavior is representative of their daytime space use. Therefore, our non-breeding locations, which were taken while individuals were presumably roosting, are representative of their daytime space use. KDEs were calculated using least-square cross validation to estimate bandwidth [7,23] with the ks package[24] in program R[25]. The migration distance between breeding and non-breeding territories

was calculated as the Euclidean distance between the two locations. Environmental attributes were annotated to location data used Env-DATA Track Annotation Service tool[5]. Inverse distance weighted interpolation methods were used when available to estimate the value of each environmental attribute. We considered elevation (m asl), distance to coastline (km), and high vegetation cover (%, includes trees, forest, woodland) as physical site characteristics of non-breeding territories. Environmental variables such as air and soil temperature (°C), normal difference vegetation index (NDVI) and the change in NDVI values from October (early non-breeding) to March (prior to spring departure) were characterized as environmental variables. We used the change in NDVI from October to March to quantify vegetation change throughout the non-breeding period and the most recent land cover classification (2012) of each non-breeding location as potential indicators of habitat quality. We used t-tests to compare migration distances and environmental variables of non-breeding territories between the two populations. We conducted t-tests in a Bayesian framework with vague priors. We ran t-tests in JAGS (Just Another Gibbs Sampler)[26] called from R2jags[27] using three chains of 50,000 iterations each. Every 10th sample was drawn from the posterior distribution after discarding the first 10,000 iterations as a burn-in resulting in 12,000 samples from the posterior distribution from which we drew inference.

Delineating population boundaries

To determine whether the number of individuals sampled provided reliable estimates of the population boundaries we generated population boundaries for all possible combinations of individuals ranging from three to ten individuals. Because our sample size from HBEF was limited for determining the population boundary asymptote, we compared our estimated population boundary to geolocator estimates (n=21) from the same breeding population[12]. KDEs

were calculated using least-square cross validation to estimate bandwidth[7,23] with the ks package[24] in program R[25].

Migratory connectivity

We calculated a breeding and non-breeding distance matrix based on the capture locations during the breeding season and the final location obtained from the GPS tags during the non-breeding season (range 21 October 2013: 7 April 2014). We conducted three Mantel tests, one where we included all individuals, and one for each capture location. We constructed breeding and non-breeding distance matrices using the dist function in program R. We ran Mantel tests on the breeding and non-breeding distance matrices using 10,000 permutations to assess the *P*-value of r_M using the mantel.rtest function in the ade4 package[28] in program R.

GPS tags were scheduled to take location fixes at 28 day intervals. Thus, we obtained locations on 23 September 2013 coinciding with autumn migration[12] and autumnal equinox, the period when latitudinal estimates derived via geolocators are unreliable. We calculated latitudinal differences between breeding territories and the location of individuals on 23 September. We used that distance as a proxy for migration timing. We used a t-test to determine if migration timing differed between the two populations.

RESULTS

Thirteen of the 15 tags (87%) collected all 10 location points as scheduled, one collected 7 points (6%) and the other collected 6 points (6%) but all tags took location fixes at the individuals final non-breeding location (no movement between consecutive fixes - 28 days).

Attributes of non-breeding territories

Individuals breeding at HBEF (282.66m, CI: 195.68-367.88) wintered at higher elevations than individuals breeding at JBWS (38.75m, CI: 16.33-45.47; mean difference = 243.91, CI: 154.61-332.13).

Migratory connectivity-

Migration timing did not differ significantly between the two capture locations (mean = 5.49°, CI: -3.08-11.65) despite having strong connectivity and non-overlapping population boundaries. However, individuals captured at JBWS tended to be further from their breeding territories on 23 September than birds breeding at HBEF (Fig. 2). All but two individuals (20%) captured at JBWS had left breeding territories and were either migrating (20%, n=2) or had arrived at their non-breeding territories (40% n=6) by 23 September. In contrast, all individuals captured at HBEF had left their breeding territories (100%, n=5) but had not yet reached their final non-breeding location in the tropics.

Delineating population boundaries

The non-breeding population boundary size for JBWS began to reach an asymptote around our sample size of 10 individuals (Fig. S2). The population boundary asymptote was not achieved with a sample size of five individuals from HBEF, however the population boundary fell entirely within the geolocator estimates of 21 individuals from the same breeding population.

LITERATURE CITED

21. Naef-Daenzer, B. An allometric function to fit leg-loop harnesses to terrestrial birds. *Journal of Avian Biology* **38,** 404–407 (2007).

22. Hallworth, M. T. The influence of migratory connectivity and seasonal interactions on individual- and population-level dynamics of a long distance migratory songbird. Ph.D. Dissertation, George Mason University, Fairfax, VA. 22032. (2014).

23. Barg, J. J., Jones, J. & Robertson, R. J. Describing breeding territories of migratory passerines: suggestions for sampling, choice of estimator, and delineation of core areas. *Journal of Animal Ecology* **74,** 139–149 (2005).

24. Duong, T. ks: Kernel smoothing. R package version 1.8. 2. *See http://CRAN. R-project. org/package= ks* (2011).

25. R Core Team. *R: A language and environment for statistical computing.* (R Foundation for Statistical Computing, 2013). at <http;//www.R-project.org/>

26. Plummer, M. *JAGS: A program for analysis of Bayesian graphical models using Gibbs sampling*. (2003).

27. Su, Y.-S. & Yajima, Masanao. R2jags: A package for running jags from R. *R package version 0.03-08* (2012). at <http://CRAN. R-project. org/package= R2jags>

28. Dray, S. & Dufour, A. B. The ade4 package: implementing the duality diagram for ecologists. *Journal of Statistical Software.* **22,** 1–20 (2007).

Table S1. The source, spatial resolution and measurement unit of non-breeding territory attributes annotated to location data using the Env-DATA Track Annotation Service tool from movebank.org[5].

Non-Breeding Attribute	Units	Spatial Resolution	Source
Physical			
Elevation	m	0.00028°	https://lpdaac.usgs.gov/products/aster_products_table/astgtm
Distance to coastline	km	0.04°	http://oceancolor.gsfc.nasa.gov/DOCS/DistFromCoast/
Vegetation Cover (High)	na	0.75°	http://apps.ecmwf.int/datasets/data/interim_full_invariant/
Environmental			
Land Cover	na	0.083°	https://glcf.umd.edu/data/lc/
NDVI	na	0.1°	ftp://neoftp.sci.gsfc.nasa.gov/rgb/MOD13A2_E_NDVI/
Oct-Mar	na	0.1°	ftp://neoftp.sci.gsfc.nasa.gov/rgb/MOD13A2_E_NDVI/
Air Temp	°C	0.75°	http://apps.ecmwf.int/datasets/data/interim_full_daily/
Soil Temp	°C	0.75°	http://apps.ecmwf.int/datasets/data/interim_full_daily/

Table S2. The number of GPS locations taken for each Ovenbird with a GPS tag breeding in the Hubbard Brook Experimental Forest, NH (HBEF) and Jug Bay Wetland Sanctuary, MD (JBWS). The number of locations taken on each date is shown and the dates when locations were taken during the non-breeding season are indicated in the Non-breeding column.

	2013							2014					
Individual	July 1	July 29	Aug. 26	Sept. 23	Oct. 21	Nov. 18	Dec. 16	Jan. 13	Feb.10	Mar.10	Apr.7	Total Locations	Non-breeding
HBEF													
45043	1	1	1	1	1	2	1	1	1			10*	18 Nov – 10 Feb
45050	1	1	1	1	1	1		1	1	1	1	10	18 Nov – 7 Apr
45056	1	1	1	1	1	1	1	1	1	1		10	21 Oct – 10 Mar
45058	1	1	1	1	2							6	21 Oct – 21 Oct
45068	1	1	1	1	1	1	1					7	21 Oct – 16 Dec
JBWS													
91146	1	1	1	1	1	1	1	1	1	1		10	21 Oct – 10 Mar
45005	1	1	2	1	1	1	1	2				10*	21 Oct – 13 Jan
45008	1	1	1	1	1	1		1	2	1		10*	23 Sept – 10 Mar
45011	1	1	1	1	1	1	1	1	2			10*	23 Sept – 10 Feb
45013	1	1	1	1	2	1	1	2				10*	21 Oct – 13 Jan
45018	1	1	1	1	1	2	1	1	1			10*	21 Oct – 10 Feb
45020	1	1	1	1		1	1	1	1	1	1	10	23 Sept – 7 Apr
45026	1	1	1	2	1	2	1	1				10*	23 Sept – 13 Jan
45027	1	1	1	1	1	1	1	1	1	1		10	23 Sept – 10 Mar
45028	1	1	1	1	1	1	1	1	1	1		10	23 Sept – 10 Mar

* indicates that two points were taken on the same day because of a software malfunction

Figure S1. The latitudinal distance Ovenbirds were from breeding territories in New Hampshire (HBEF) and Maryland (JBWS) on 23 September 2013. The mean difference (solid line) and 95% CI (dashed lines) between the populations are also shown.

Figure S2. Non-breeding population boundary size as a function of the number of individuals with GPS tags captured at Hubbard Brook Experimental Forest, NH (HBEF) and Jug Bay Wetland Sanctuary, MD (JBWS). The mean ± SE are shown. No standard error is shown for the final size because only one population boundary was created using all individuals.

Figure S1.

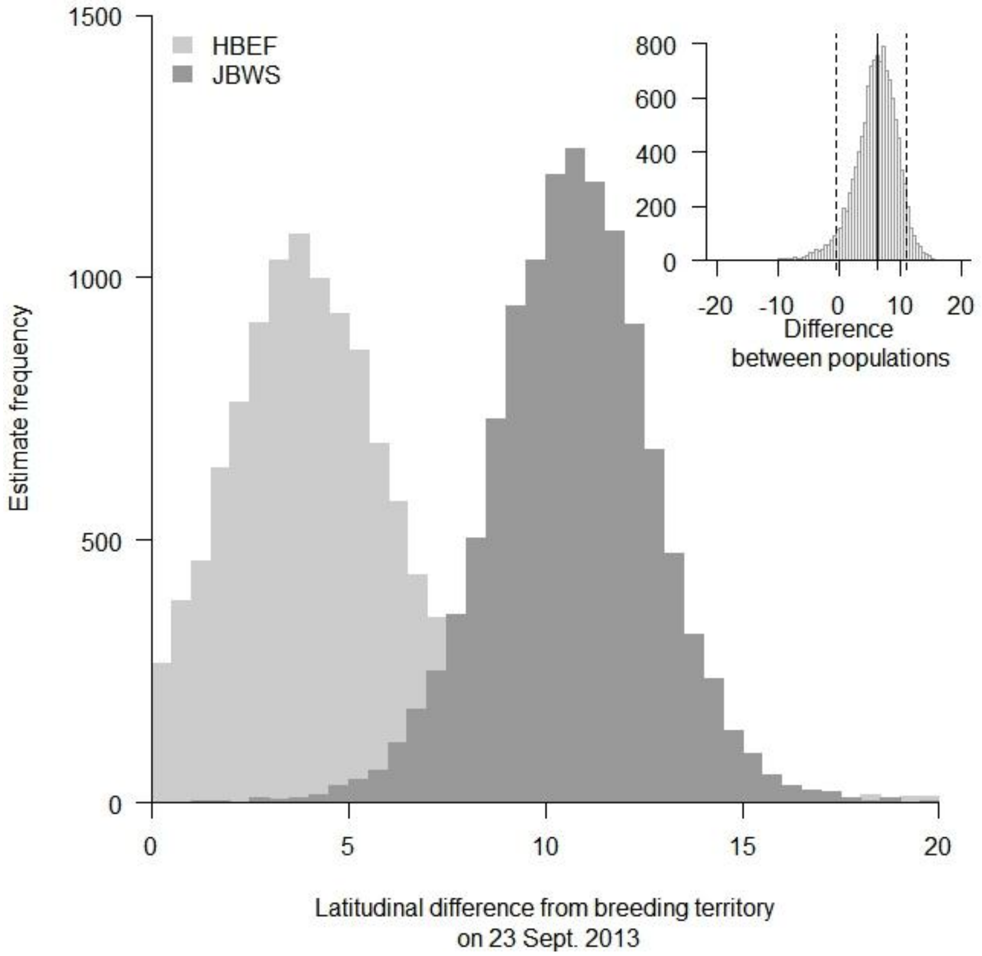

Figure S1. The latitudinal distance Ovenbirds were from breeding territories in New Hampshire (HBEF) and Maryland (JBWS) on 23 September 2013. The mean difference (solid line) and 95% CI (dashed lines) between the populations are also shown.

Figure S2.

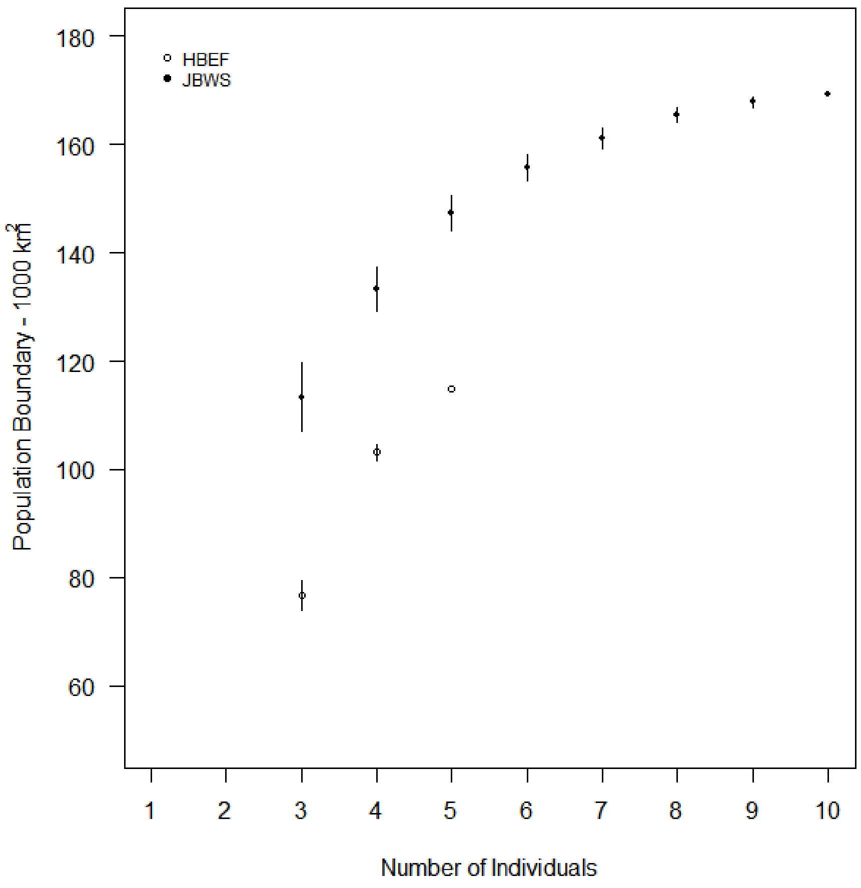

Figure S2. Non-breeding population boundary size as a function of the number of individuals with GPS tags captured at Hubbard Brook Experimental Forest, NH (HBEF) and Jug Bay Wetland Sanctuary, MD (JBWS). The mean ± SE are shown. No standard error is shown for the final size because only one population boundary was created using all individuals.
